# Responses of nutrient utilization, rumen fermentation and microorganisms to different roughage of dairy buffaloes

**DOI:** 10.1186/s12866-024-03342-0

**Published:** 2024-05-29

**Authors:** Shichun He, Ruiyun Zhang, Rongjiao Wang, Dongwang Wu, Sifan Dai, Zibei Wang, Tao Chen, Huaming Mao, Qing Li

**Affiliations:** 1 Yunnan Provincial Key Laboratory of Animal Nutrition and Feed Science, Faculty of Animal Science and Technology, Yunnan Agricultural University, Kunming, 650201 China; 2 Institute of Animal and Veterinary Medicine, Panzhihua Academy of Agricultural and Forestry Sciences, Panzhihua, 617000 China; 3 Animal Husbandry station in Mangshi, Dehong Prefecture, Mangshi, Yunnan, 678400 China

**Keywords:** Buffaloes, Roughage, Digestibility, Ruminal fermentation parameters, Ruminal microorganisms

## Abstract

Dairy buffaloes are typically fed a high-forage, low-quality diet with high fiber. These conditions result in an inherent energy and protein inefficiency. In order to make full and rational use of feed resources and improve the production level and breeding efficiency of dairy buffaloes, the effects of various roughages on nutrient digestibility, ruminal fermentation parameters, and microorganisms in dairy buffaloes were studied in this experiment. Three ternary hybrid buffaloes, with an average body weight of 365 ± 22.1 kg, were selected and fitted with permanent rumen fistulas. They were fed six different diets, each consisting of 1 kg concentrate supplement and one of six types of roughage, including alfalfa hay (A diet), oat hay (O diet), whole corn silage (W diet), king grass (K diet), sugarcane shoot silage (S diet), and rice straw hay (R diet) according to an incomplete Latin square design of 3 × 6, respectively. The pre-feeding period of each period was 12 d. From day 13 to 15 was the official experimental period. During the prefeeding period, free feed intake for each roughage was determined, and during the experiment, the roughage was fed at 90% of the voluntary feed intake. Digestion and metabolism tests were carried out using the total manure collection method to determine the feed intake and fecal output of each buffalo, and to collect feed and fecal samples for chemical analysis. On day 15, rumen fluid samples were collected two hours after morning feeding to determine rumen fermentation parameters and bacterial 16 S rRNA high-throughput sequencing was performed. The results showed that DM and OM digestibility were greatest for the W diet and lowest for the S diet. The rumen pH of the O diet was significantly greater than that of the W diet. The concentration of rumen fluid NH_3_-N (mg/dL) increased with increased CP content. The concentration of total volatile fatty acids (mmol/L) in the rumen decreased with increased NDF content but increased with increased NFC content. The relative abundances of Bacteroidetes, Firmicutes, and Spirochaetes were 57.03-74.84%, 14.29-21.86%, and 0.44-1.43% in the different quality roughage groups. Bacteroidetes were mainly *Prevotellaceae*1 and *Rikenellaceae* RC_gut_group with relative abundances of 30.17-45.75% and 3.23-7.82%. The relative abundance of Patescibacteria and Spirochaetes decreased with increasing roughage quality. These results provide a theoretical and practical basis for evaluating the nutritional value of dairy buffalo feed, utilizing feed resources, matching rations, feeding scientifically, and protecting animal health.

## Introduction

Buffaloes have special digestion and metabolic physiology compared with ordinary cattle, with a greater capacity to digest roughage due to the rumen microbial composition [1–3]. Under the same diet conditions, the intake of dry matter, crude protein, total digestible nutrients, and metabolizable energy, as well as the digestibility of neutral detergent fiber were significantly higher in buffalo than in Brahman cattle [4]. Additionally, the levels of fiber digestion, nitrogen cycling, and rumen ammonia nitrogen related to rumen fermentation were higher in buffalo compared with cattle [5]. Sirohi et al. have confirmed that the number of cellulose-degrading bacteria, Bacillus filiformis succinogenes and Ruminococcus albus were significantly higher in buffalo than in cattle [6]. A complete metagenomic sequence was conducted on buffalo and revealed that 64–84% of the bacterial coding sequences were present in the buffalo rumen, with the dominant bacteria at the phylum level being Bacteroidetes (52–64%), Firmicutes (18–22%), and Proteobacteria (10–15%) [7]. Boonsaen [8] identified a strain OS14 in buffalo that exhibited higher than degradation ability towards straw and other tropical herbage compared to other strains and demonstrated an enhanced digestion effect on herbage when cocultured with selenomonas ruminatium. The digestibility of fiber in buffalo is higher than that in other ruminants, which may be due to the difference in the fermentation products caused by rumen microorganisms in buffalo that affect fiber digestion. Therefore, the differences in the species and numbers of bacteria, fungi, and protozoa in the rumen of buffalo and other ruminants can be used to explain the variations in their digestibility.

There are many kinds of rumen microorganisms, such as bacteria, fungi, protozoa and archaea. Forages are first degraded and fermented by rumen microorganisms, and then absorbed and utilized by the animal’s secretory enzymes [9]. High-quality forages (e.g., alfalfa hay, oat hay and whole corn silage) improve feed utilization and high lactation performance. Low-quality forages (e.g., king grass, sugarcane shoot silage and rice straw hay) often decreases the metabolizable energy content of the diets because of their poor palatability, low digestibility, and low content of crude protein and non-fiber carbohydrate [10]. Numerous studies have confirmed that the microbial composition of the rumen varies with changes in diet. Nutrients in the diet are first converted into small molecules that can be utilized by the body through fermentation of rumen microorganisms, and then enter various systems to perform their functions, which has a significant impact on the production performance of cattle [11, 12]. As shown in previous studies, different feed compositions can alter the relative abundance of the rumen Bacteroidetes, Firmicutes and Proteobacteria, synergistic effects of different rumen microorganisms on rumen metabolites and microbial functions [13, 14].

Providing scientific and reasonable diets is the key to improve the production level and breeding efficiency of dairy buffaloes. This study aimed to investigate the effects of different roughages on nutrient digestibility, rumen fermentation and microorganisms in dairy buffaloes. The results have important practical guidance significance for scientific and rational utilization of various feed resources, improvement of production level, and breeding efficiency of dairy buffaloes.

## Materials and methods

### Animals and experimental design

The experiment was conducted in the Dehong Mincheng Breeding Professional Cooperative from August to November 2020. The experimental site is a typical southern subtropical climate in the low heat basin climate, with an altitude of 1125.7 m, the average annual temperature of 19.5 ℃, the annual sunshine ranges from 2000 to 2452 h, and the annual rainfall ranges from 1300 to 1653 mm. Three ternary hybrid buffaloes with similar body weight (365 ± 22.1 kg) and aged around 2.5 years old were selected. A permanent rumen fistula was placed in September 2019. The inner diameter of the fistula was 10 cm. Preparations for the feeding experiment were made in July 2020. A 3 × 6 incomplete Latin design was used for this experiment. They were fed six different diets, each consisting of 1 kg concentrate supplement containing 24.02% crude protein (composition of the concentrate is shown in Table 1) and one of six types of roughage, including alfalfa hay (A diet), oat hay (O diet), whole corn silage (W diet), king grass (K diet), sugarcane shoot silage (S diet), and rice straw hay (R diet). Phase 6 trials were conducted. At the end of each trial period, the roughage is gradually changed each day until the switch from one diet to the other is completed in 7 days. The pre-feeding period of each period was 12 d. From day 13 to 15 was the official experimental period. During the prefeeding period, free feed intake for each roughage was determined, and during the experiment, the roughage was fed at 90% of the free feed intake. Digestion tests were carried out using the total manure collection method to determine the feed intake and fecal output of each buffalo, and to collect feed and fecal samples for chemical analysis. Concentrates and some roughage are mixed and fed together. This ensures that all concentrates are consumed each day. The forage was divided into two equal parts and fed at 8:00 am and 5:00 pm. Each test dairy buffalo was individually tethered and had a separate trough and water sources.

**Table 1:** Formula of concentrate supplement for cattle

Raw material	Corn	Soybean meal	Cottonseed meal	Corn gluten feed	Rice bran	Wheat	Rapeseed meal	Soybean hull	Bran	Rock fine	Dibasic Calcium Phosphate	Premix	Slow-release urea	Sodium chloride
%	7.00	12.5	4.30	10.0	18.0	5.50	8.50	10.0	16.5	1.70	1.50	2.00	1.50	1.00

### Sample collection

The feed intake of each dairy buffalo was recorded daily during the trial period. Samples of each diet were collected on three days during the trial period, dried at 65 °C for nutrient analysis. Using the total feces collection method, feces were collected from each buffalo during the trial period, dried at 65 °C for nutrient analysis. On day 15, rumen fluid samples were collected through a rumen fistula 2 h after the morning feed.

### Chemical analysis

The collected samples were crushed in a grinder and analyzed for dry matter (DM), organic matter (OM), neutral detergent fiber (NDF), acid detergent fiber (ADF), crude protein (CP), non-fiber carbohydrate (NFC), acid detergent lignin (ADL), ether extract (EE), calcium (Ca), phosphorus (P).

Rumen fluid pH was measured directly using a PHS-3 C pH meter after rumen fluid collection. NH_3_-N was determined by the phenol-hypochlorous acid colorimetric method. 40 µL of supernatant was taken into a labeled test tube after rumen fluid centrifugation at 12,000 × g for 20 min, 2.5 mL of phenol chromogenic agent and 2.0 mL of sodium hypochlorite reagent were added, then completely mixed by swirling and placed in a water bath at 37 ℃ for 30 min. Colorimetric analysis of the supernatant was performed using a visible spectrophotometer at a wavelength of 550 nm. Replace the rumen fluid with NH_4_Cl standard solution and repeat the above steps, then plot the standard curve. The NH_3_-N concentration was calculated from the regression equation, colorimetric results and standard curve.

Volatile fatty acids (VFA) were determined by gas chromatography-mass spectrometry. Take sample 20µL into EP tubes, add 380µL ddH_2_O, vortex oscillated for 30 s, and follow add 100µL 15% phosphoric acid, 20µL 75 mg/mL internal standard solution and ether 280µL homogenized for 1 min. Centrifuge for 10 min at 12,000 rpm, 4℃. The chromatographic column utilized an Agilent HP-INNOWAX capillary column (30 m*0.25mmID*0.25 μm), injection volume of 1µL, shunt ratio of 10:1. Inletion source and transmission line temperatures were 250 ℃, 230 ℃ and 250 ℃. The initial temperature of the programmed temperature increase was 90 °C, then 10 °C/min to 120 °C, then 5 °C/min to 150 °C, and finally 25 °C/min to 250 °C for 2 min. The carrier gas was helium and the carrier gas flow rate was 1.0 mL/min.

### DNA extraction and sequencing

DNA was purified using the Zymo Research BIOMICS DNA Microprep Kit (Cat# D4301) and DNA integrity was checked by 1% agarose electrophoresis, followed by nucleic acid concentration assay using a Tecan F200. The V4 region of the bacterial 16S ribosomal RNA genes were amplified by PCR (94°C for 1 min, followed by 30 cycles at 94°C for 20 s, 54°C for 30 s, and 72°C for 30 s and a final extension at 72°C for 10 min), using indexes and adaptor-linked universal primers (515F: 5’-GTGYCAGCMGCCGCGGTAA-3’; 806R: 5’-GGACTACHVGGGTWTCTAAT-3’). PCR reactions were performed in 30 µL mixtures containing 15 µL of 2 × KAPA Library Amplification Ready Mix, 1 µL of each primer (10 µM), and 50 ng of template DNA and ddH_2_O. The PCR product was mixed with 6× sample buffer, followed by electrophoresis of the target fragments on a 2% agarose gel. Using the ZymocleanGel Recovery Kit (D4008) were recovered the target bands and quantified using a Qubit@ 2.0 Fluorometer (Thermo Scientific). Library construction used the NEW ENGLAND BioLabs NEBNext Ultra II DNA Library Prep Kit for Illumina (NEB#E7645L). PE250 sequencing method was adopted, and Illumina Hiseq Rapid SBS Kit v2(FC-402-4023 500 Cycle) was used as a sequencing kit. The original 16 S rRNA data were available at the National Center for Biotechnology Information (NCBI) SRA database with accession number PRJNA1001128.

### Data analysis

Apparent digestibility = (nutrient intake - nutrient excretion)/nutrient intake×100% [15].

Latin square design ANOVA based on SPSS was applied to compare dietary nutrient composition, digestibility and rumen fermentation parameters between the different groups, and the differences were considered statistically significant at *P* < 0.05.

The raw data of each sample was first obtained by splitting according to the barcode, and the barcode and primers were removed, followed by splicing double-ended sequences data using FLASH [16]. QIIME (v1.9.0) [17] is then used for quality control by filtering out sequences with an average quality of less than 25, removing sequences less than 200 bp in length, removing sequences with more than 2 fuzzy bases (N), and removing chimeras using the uchime algorithm and the gold database in order to obtain a valid tag. Using UPARSE [18] algorithm to perform OTU clustering at 97% consistency level. The sequence with the highest frequency in each OTU was selected as the representative sequence of OTU for annotation analysis using UCLUST [19] classification method and SILVA (silva 132) [20] database. Multiple alignments of representative sequences are performed using PyNAST [21]. Alpha diversity being made in R. The PD index is calculated using the Picante package, and the other indices are calculated using the Vegan package. The Wilcoxon rank sum test was performed using the wilcox. test function of the stats package, and the Kruskal-Wallis rank sum test was performed using the Kruskal.test function for both groups. Multiple comparisons are performed using the agricolae package. Beta diversity is being made in R (3.6.0). Unifrac distances were calculated using the GuniFrac package, and Bray-Curtis and Jaccard distances were calculated using the vegdits function of the Vegan package. PCA analysis with the vegan package. The LEfSe software was utilized for differential species analysis.

## Results

### Nutrient digestibility of each experimental diet

According to the actual feed intake of roughage and concentrate during each trial period, we obtained the nutrient content of the diets for different experimental groups, as shown in Table 2. The A diet had the highest CP content, while the R diet had the lowest. The A, O, and W diets had lower NDF content compared to the other three diets (*P* < 0.05) and there were significant differences among the three diets (*P* < 0.05). The A and W diets had significantly higher NFC content than the K and R diets (*P* < 0.05).

**Table 2:** The nutritional composition of different diets (dry matter basis)

Item	A diet	O diet	W diet	K diet	S diet	*R* diet
Concentrate intake(kg)	0.88	0.88	0.88	0.88	0.88	0.88
Roughage intake(kg)	6.78 ± 1.66^ab^	7.02 ± 0.60^ab^	8.25 ± 1.76^a^	5.97 ± 0.75^bc^	7.31 ± 0.83^ab^	4.78 ± 0.62^c^
Total intake (kg)	7.66 ± 1.66^ab^	7.90 ± 0.59^ab^	9.13 ± 1.75^a^	6.85 ± 0.75^ab^	8.19 ± 0.82^ab^	5.67 ± 0.62^b^
Roughage intake / Total intake(%)	88.51	88.86	90.36	87.15	89.26	84.30
DM(%)	89.2 ± 2.15^a^	85.8 ± 0.43^b^	30.3 ± 2.37^d^	24.7 ± 1.98^f^	30.0 ± 1.65^e^	82.4 ± 1.96^c^
OM(%)	90.9 ± 0.42^cd^	92.0 ± 1.39^bc^	94.3 ± 0.73^a^	89.0 ± 2.02^cd^	93.5 ± 0.14^ab^	85.8 ± 0.59^e^
CP(%)	17.0 ± 0.40^a^	8.71 ± 0.11^c^	9.46 ± 0.24^c^	10.6 ± 1.79^b^	7.49 ± 0.44^d^	6.66 ± 0.50^d^
EE(%)	1.35 ± 0.26^ab^	1.65 ± 0.53^ab^	2.11 ± 1.02^ab^	2.18 ± 0.57^ab^	2.42 ± 0.36^a^	1.11 ± 0.09^b^
Ca(%)	1.39 ± 0.09^a^	0.53 ± 0.09^cd^	0.59 ± 0.10^bc^	0.71 ± 0.02^b^	0.58 ± 0.03^bc^	0.47 ± 0.05^d^
P(%)	0.25 ± 0.02^c^	0.23 ± 0.03^c^	0.27 ± 0.01^b^	0.32 ± 0.01^a^	0.21 ± 0.02^d^	0.13 ± 0.02^e^
NDF(%)	38.4 ± 1.92^e^	52.7 ± 2.20^c^	46.4 ± 0.13^d^	62.8 ± 3.19^b^	66.4 ± 1.68^ab^	67.3 ± 1.11^a^
ADF(%)	31.1 ± 1.08^c^	33.9 ± 1.60^c^	32.5 ± 3.37^c^	45.0 ± 3.51^b^	42.4 ± 0.75^b^	48.9 ± 1.56^a^
ADL(%)	6.69 ± 0.15^a^	4.76 ± 0.37^bc^	4.33 ± 0.37^c^	5.63 ± 0.70^ab^	5.85 ± 0.17^ab^	6.42 ± 0.72^b^
NFC(%)	34.2 ± 1.77^a^	29.2 ± 3.75^ab^	36.4 ± 0.33^a^	13.6 ± 0.18^c^	17.3 ± 1.15^bc^	10.9 ± 1.26^c^

Note: Means within a row with different superscripts differ significantly at *P* < 0.05. A: Alfalfa hay. O: Oat hay. W: Whole corn silage. K: King grass. S: Sugarcane shoot silage. R: Rice straw hay. DM: Dry Matter. OM: Organic Matter. CP: Crude Protein. EE: Ether Extract. Ca: Calcium. P: Phosphorus. NDF: Neutral Detergent Fiber. ADF: Acid Detergent Fiber. ADL: Acid Detergent Lignin. NFC: Non-fiber Carbohydrates

As shown in Table 3, The DM of W diet was significantly higher than that of the S diet (*P* < 0.05), and 10.96% higher than that of the S diet; however, no significant difference was found among other diets (*P* > 0.05). The OM of W diet was also significantly higher than that of the S diet (*P* < 0.05), with a difference of 11.4%. Nevertheless, there was no significant difference in OM among other diets (*P* > 0.05). The CP of K diet was higher than that in the S diet (*P* < 0.05)and the R diet (*P* < 0.05), but it did not show a significant difference with other diets (*P* > 0.05). The EE of A diet, S diet and K diet were significantly higher than that of the R diet (*P* < 0.05). The NDF of A diet was significantly lower than that of the K diet and R diet (*P* < 0.05), while there was no significant difference among other diets (*P* > 0.05). The ADF of R diet and K diet was significantly higher than that of the A diet (*P* < 0.05). The ADL of R diet was significantly higher than that of the A diet, O diet, and S diet (*P* < 0.05). The NFC of R diet was significantly higher than that of the S diet (*P* < 0.05).

**Table 3:** Comparison of the digestibility of conventional nutrient composition and Van’s detergent fiber in dairy buffaloes

Item	A diet	O diet	W diet	K diet	S diet	*R* diet
DM(%)	61.5 ± 1.02^ab^	61.3 ± 1.58^ab^	65.0 ± 6.50^a^	59.0 ± 8.83^ab^	54.1 ± 0.37^b^	55.6 ± 2.82^ab^
OM(%)	63.1 ± 1.44^ab^	64.3 ± 1.74^ab^	67.2 ± 6.21^a^	61.1 ± 8.41^ab^	55.8 ± 0.44^b^	61.2 ± 3.87^ab^
CP(%)	70.1 ± 8.25^a^	59.4 ± 2.25^ab^	60.0 ± 7.30^ab^	73.4 ± 6.73^a^	44.7 ± 6.16^b^	51.5 ± 10.66^b^
EE(%)	73.8 ± 5.27^a^	67.6 ± 4.99^ab^	65.8 ± 13.24^ab^	70.8 ± 2.80^a^	71.8 ± 10.70^a^	53.8 ± 8.84^b^
Ca(%)	35.2 ± 3.65^ab^	18.7 ± 15.24^bc^	45.6 ± 4.34^a^	30.0 ± 6.54^ab^	17.8 ± 9.14^c^	18.7 ± 7.65^bc^
P(%)	25.2 ± 9.98^ab^	24.3 ± 11.65^ab^	33.0 ± 7.29^a^	40.4 ± 15.25^a^	20.8 ± 8.38^ab^	7.43 ± 7.04^b^
NDF(%)	36.2 ± 3.68^b^	47.7 ± 6.18^ab^	49.0 ± 9.98^ab^	53.5 ± 8.54^a^	49.0 ± 1.81^ab^	56.4 ± 3.31^a^
ADF(%)	39.5 ± 4.51^b^	42.6 ± 6.01^ab^	47.3 ± 9.85^ab^	51.2 ± 7.50^a^	48.3 ± 2.58^ab^	56.0 ± 10.16^a^
ADL(%)	10.6 ± 1.58^b^	8.79 ± 0.50^b^	13.9 ± 2.74^ab^	17.0 ± 6.51^ab^	12.6 ± 0.11^b^	26.3 ± 8.24^a^
NFC(%)	89.6 ± 2.94^ab^	95.0 ± 3.52^ab^	92.1 ± 3.84^ab^	85.5 ± 10.11^ab^	84.8 ± 5.83^b^	98.0 ± 4.50^a^

Note: Means within a row with different superscripts differ significantly at *P* < 0.05. A: Alfalfa hay. O: Oat hay. W: Whole corn silage. K: King grass. S: Sugarcane shoot silage. R: Rice straw hay. DM: Dry Matter. OM: Organic Matter. CP: Crude Protein. EE: Ether Extract. Ca: Calcium. P: Phosphorus. NDF: Neutral Detergent Fiber. ADF: Acid Detergent Fiber. ADL: Acid Detergent Lignin. NFC: Non-fiber Carbohydrates

### Rumen fermentation parameters

Table 4 shows that rumen pH values of the O diet with different roughage diets were significantly higher than those of the W diet (*P* < 0.05), but there was no significant difference among other diets (*P* > 0.05). pH values ranged from 6.26 to 6.76, all within the normal range. The ammonia nitrogen content of the A diet and K diet was significantly higher than that of the other 4 diets, and the A diet was significantly higher than the K diet (*P* < 0.05), the K diet was significantly higher than the O diet and S diet (*P* < 0.05). the W diet was significantly higher than the O diet and R diet (*P* < 0.05). There are differences in the contents of fatty acids in 6 different diets. The TVFA of the W diet and the A diet are significantly higher than those in the R diet (*P* < 0.05), and 19.32mmol/L and 18.69mmol/L higher than those in the R diet, respectively. The proportion of acetic acid in total volatile fatty acids was 67.1–75.5%, and the higher proportion was in groups R and K diet, which was significantly higher than that in group O diet(*P* < 0.05). The proportion of propionic acid was 16.4–22.4%, and the proportion in group O diet was the highest, which was significantly higher than that in groups K, R and A diets (*P* < 0.05). The proportion of butyric acid was 5.59–8.98%, the highest in the group O diet, which was significantly higher than that in the K and R diets (*P* < 0.05). The acetic acid/propionic acid ratio was 3.05 to 4.47, and the O diet was significantly lower than that of the A diet, K diet, and R diet (*P* < 0.05), but had no significant difference with the W diet and S diet (*P* > 0.05).

**Table 4:** Comparison of rumen pH, ammonia nitrogen and the proportion of fatty acids in total volatile fatty acids in rumen in dairy buffaloes

Item	A diet	O diet	W diet	K diet	S diet	*R* diet
pH value	6.63 ± 0.17^ab^	6.76 ± 0.09^a^	6.26 ± 2.72^b^	6.43 ± 0.04^ab^	6.56 ± 0.22^ab^	6.64 ± 0.25^ab^
NH_3_-N(mg/dL)	34.02 ± 3.75^a^	10.14 ± 1.56^d^	20.30 ± 2.45^bc^	25.84 ± 6.49^b^	15.14 ± 4.43^cd^	8.57 ± 1.55^d^
Total volatile fatty acids (mmol/L)	73.85 ± 7.89^a^	64.25 ± 1.82^ab^	74.48 ± 7.43^a^	62.05 ± 7.18^ab^	69.63 ± 11.48^ab^	55.16 ± 3.30^b^
Acetic acid(%)	72.6 ± 2.41^ab^	67.1 ± 5.18^b^	70.4 ± 1.05^ab^	74.9 ± 1.70^a^	69.2 ± 1.72^ab^	75.5 ± 2.94^a^
Propionic acid(%)	16.4 ± 1.74^b^	22.4 ± 2.94^a^	19.1 ± 1.16^ab^	17.1 ± 1.29^b^	20.7 ± 0.68^ab^	17.2 ± 2.05^b^
Isobutyric acid(%)	1.18 ± 0.12^a^	0.53 ± 0.30^c^	0.89 ± 0.09^b^	0.98 ± 0.13^ab^	0.72 ± 0.09^bc^	0.69 ± 0.14^bc^
Butyric acid(%)	7.33 ± 0.31^ab^	8.98 ± 1.89^a^	6.73 ± 1.16^ab^	5.67 ± 0.60^b^	7.81 ± 1.27^ab^	5.59 ± 0.77^b^
Isovaleric acid(%)	0.85 ± 0.08^a^	0.28 ± 0.21^c^	0.76 ± 0.11^a^	0.70 ± 0.12^ab^	0.48 ± 0.08^bc^	0.45 ± 0.12^c^
Pentanoic acid(%)	1.55 ± 0.36^a^	0.71 ± 0.23^bc^	1.70 ± 0.20^a^	0.66 ± 0.09^bc^	1.00 ± 0.10^b^	0.53 ± 0.10^c^
Caproic acid(%)	0.06 ± 0.01^c^	0.04 ± 0.01^d^	0.43 ± 0.09^a^	0.03 ± 0.01^d^	0.18 ± 0.02^b^	0.04 ± 0.02^cd^
Acetic acid/ Propionic acid	4.47 ± 0.58^a^	3.05 ± 0.64^b^	3.71 ± 0.27^ab^	4.39 ± 0.41^a^	3.35 ± 0.18^ab^	4.44 ± 0.67^a^

Note: Means within a row with different superscripts differ significantly at *P* < 0.05. A: Alfalfa hay. O: Oat hay. W: Whole corn silage. K: King grass. S: Sugarcane shoot silage. R: Rice straw hay. NH_3_-N: Ammonia Nitrogen

### Rumen microbial population

#### Community richness and diversity

**Fig. 1:**
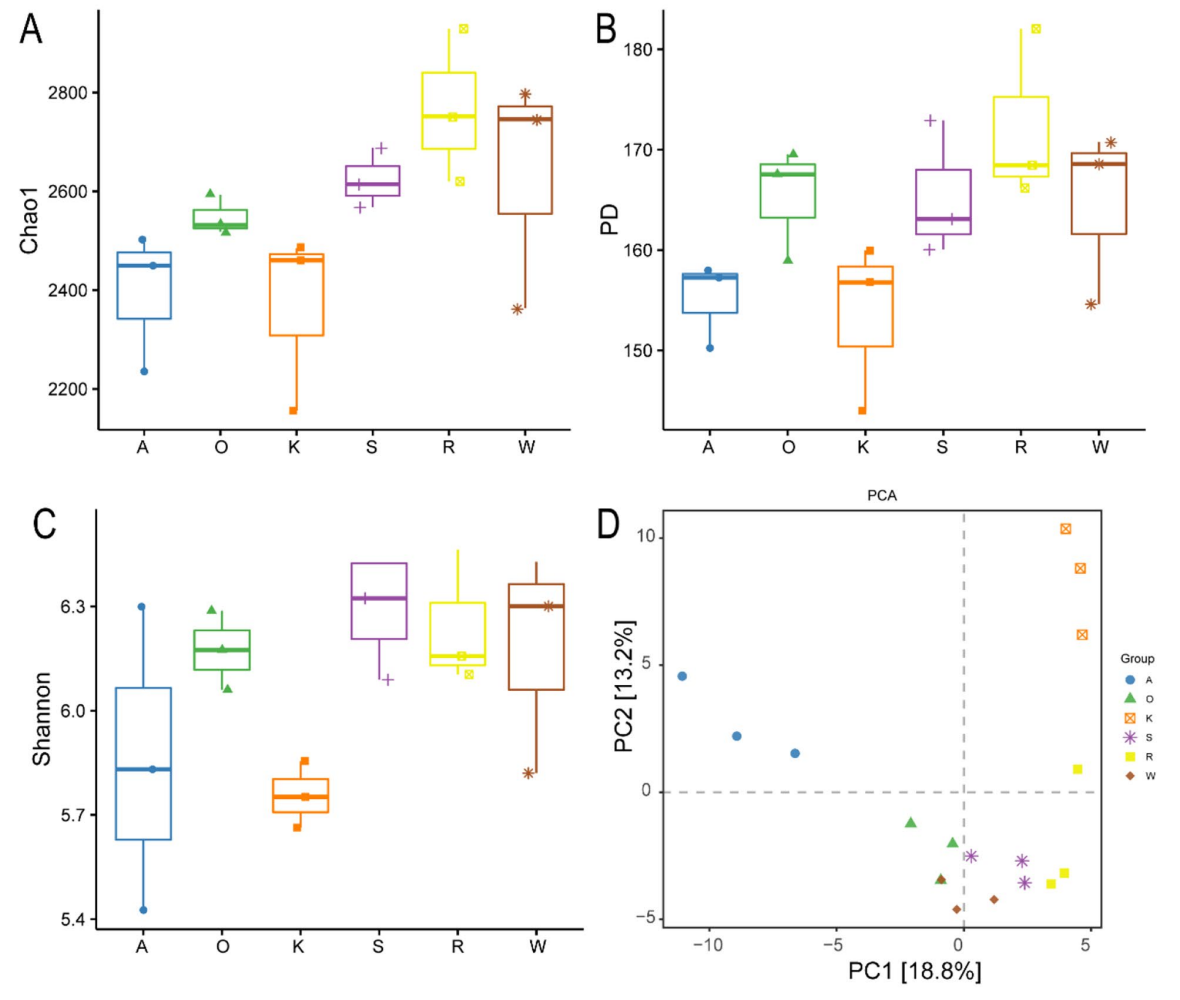
Alpha diversity and beta diversity analysis of rumen bacteria in dairy buffaloes under different diets. (**A**) Chao index. (**B**) PD. (**C**) Shannon index. (**D**) PCA. A: Alfalfa hay. O: Oat hay. W: Whole corn silage. K: King grass. S: Sugarcane shoot silage. R: Rice straw hay

The community diversity of bacteria was shown in Fig. 1. The Chao 1 index indicates community richness, which refers referring to the number of species present. The Chao1 index of R diet was significantly higher than that of the A diet and K diet. PD index reflects the differences in the preservation of the evolutionary history of the species in the sample, and the larger the index is, the greater the difference in the preservation of the evolutionary history of the species. There was no significant difference between the A diet and K diet, but it was significantly lower than the R diet (*P* < 0.05). The Shannon index reflects species diversity. The Shannon index of S diet was significantly higher than that of the K diet (*P* < 0.05). PCA analysis of dairy buffaloes with different diets separated the A diet and K diet significantly from the other four diets. The first principal component (PC1) and the second principal component (PC2) contributed 18.8% and 13.2% of the sample difference.

#### Bacterial composition at the phylum and genus level

**Fig. 2:**
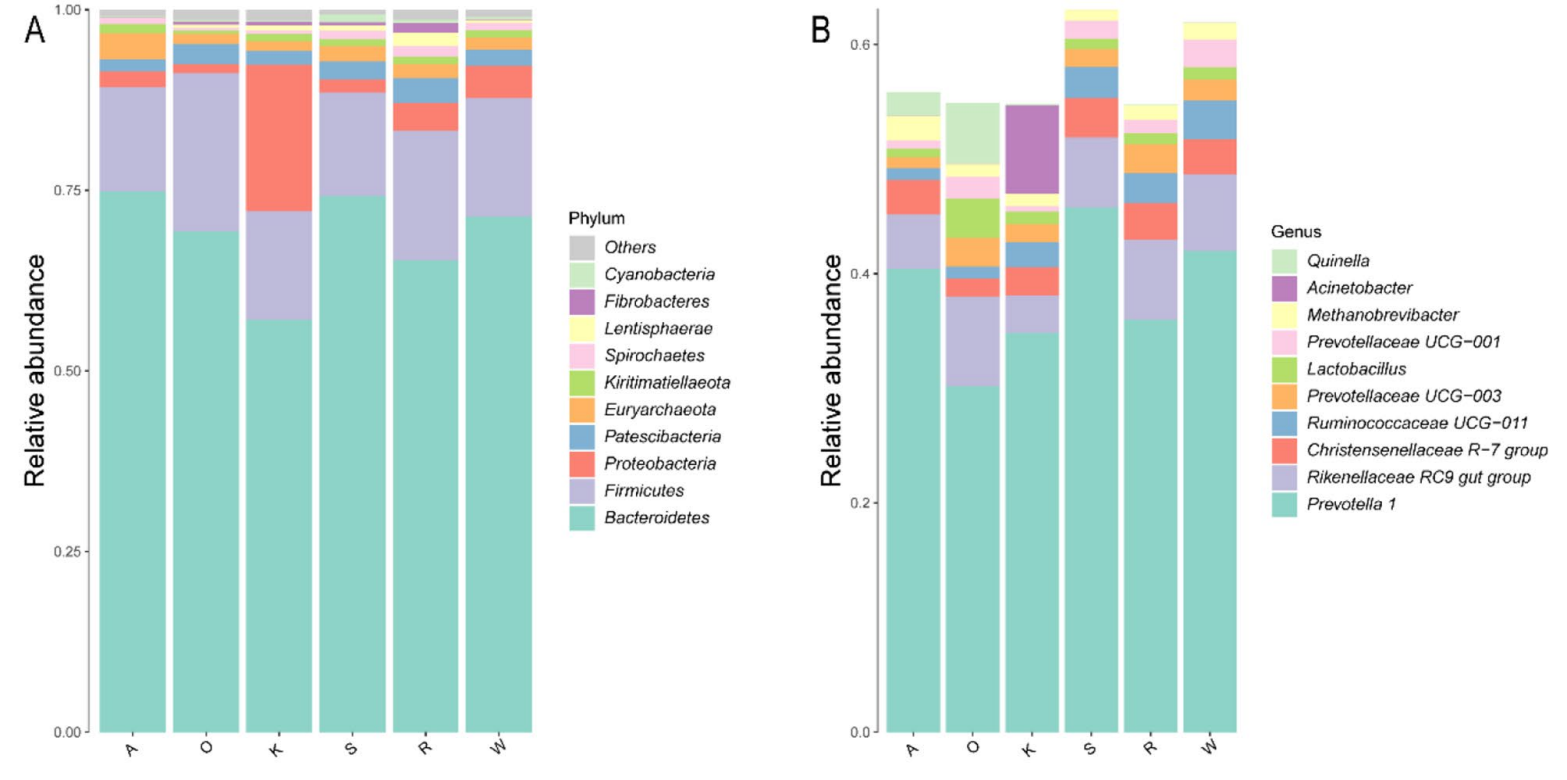
Changes of relative abundance of rumen bacterial composition in different dietary conditions. (**A**) Comparison of dominant phyla in the different diets. (**B**) Comparison of dominant genera in the different diets. A: Alfalfa hay. O: Oat hay. W: Whole corn silage. K: King grass. S: Sugarcane shoot silage. R: Rice straw hay

Fig. 2 shows the relative abundances of the top ten phyla in the rumen. Among all diet groups, Bacteroidetes and Firmicutes were the dominant bacteria. Firmicutes ranged from 14.29 to 21.86%, with the lowest values observed in the O, R, and K diets. Specifically, the percentages were 21.86%, 17.95%, 16.44%, 15.06%, 14.37%, and 14.29% for the W, K, A, S, and O diets, respectively. Proteobacteria accounted for 20.26%, 4.42%, 3.90%, 2.25%, 1.84%, and 1.26% in the K, W, R, A, S, and O diets, respectively. *Prevotella* 1 was the predominant bacterium in rumen (Fig. 2), ranging from 30.17 to 45.75%. The S diet had the highest value at 45.75%, followed by the W diet at 41.97%, the A diet at 40.44%, the R diet at 35.96%, the K diet at 34.83%, and the O diet at 37.53%. The relative abundance of the *Rikenellaceae* RC_gut_group ranged from 3.23 to 7.82%. For the O diet, R diet, the relative abundance of the *Rikenellaceae* RC_gut_group ranged from 3.23 to 7.82%, with higher values observed for the R diet and W diet, while the K diet and S diet showed lower values. The relative abundance of the *Christensenellaceae* R-7 group ranged from 1.58 to 3.46%. The abundance of *Prevotellaceae* UCG-003 ranged from 0.96 to 2.53%, with the highest values in the O diet and R diet at 2.53% and 2.49%, respectively. The abundance of *Prevotellaceae* UCG-001 ranged from 0.45 to 2.42%, with the highest value of 2.42% observed in the W diet.

#### Different species

**Fig. 3:**
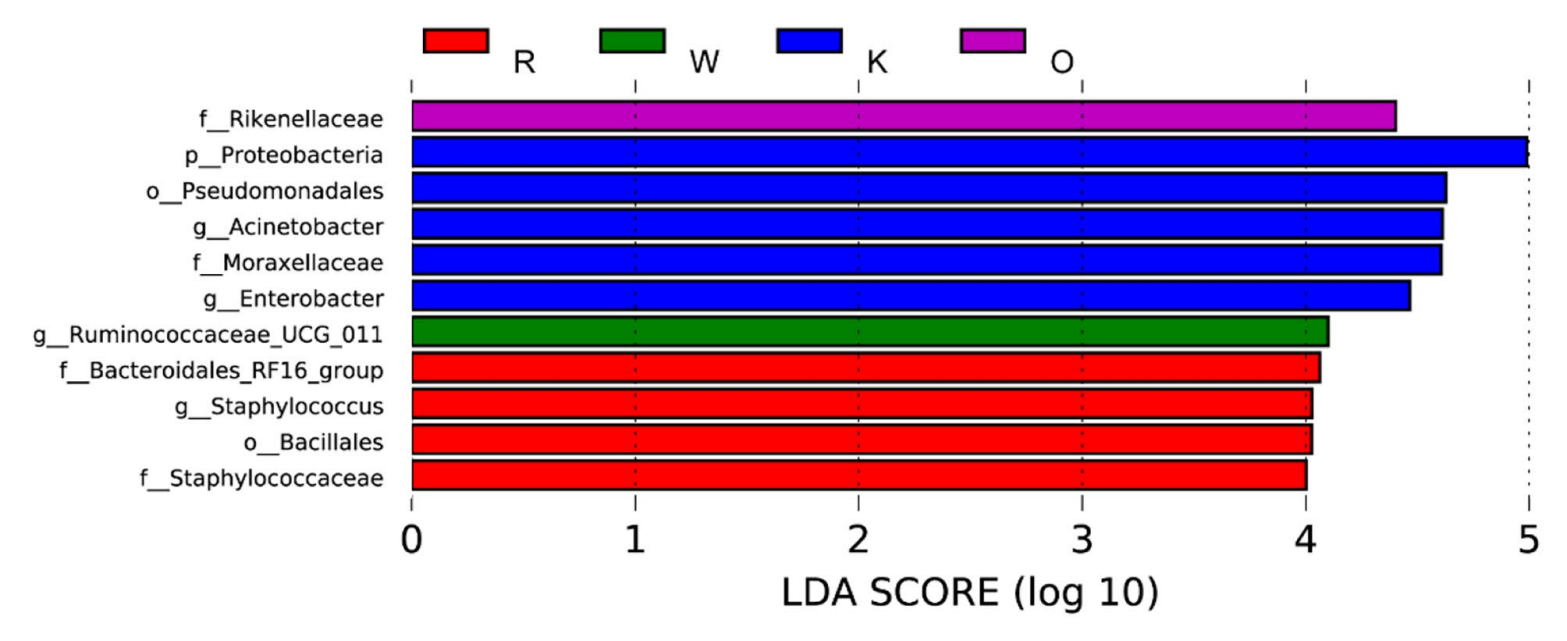
Distribution map of LDA value and evolutionary branch map of different species of rumen bacterial in dairy buffaloes under different diets. Different colors represent different groups, the K diet is shown in the blue histogram, the W diet is shown in the green histogram, the R diet is shown in the red histogram, and the O diet is shown in the purple histogram. O: Oat hay. W: Whole corn silage. K: King grass. R: Rice straw hay

LEfSe analysis was performed to identify the bacteria in six diet groups (Fig. 3). A total of 11 bacteria were listed as signature microbiota for the four groups: O diet, W diet, K diet, and R diet. The signature rumen microbiota in the K diet included Proteobacteria, Pseudomonadales, *Acinetobacter*, Moraxellaceae, and *Enterobacter*. The signature rumen microbiota in the W diet is *Ruminococcaceae*_ UCG _011. The signature rumen microbiota in the O diet is Rikenellaceae. The signature rumen microbiota in the R diet included Bacteroidales_RF16_group, *Staphylococcus*, Bacillales, and Staphylococcaceae.

## Discussion

### Effects of different roughage on nutrient digestibility of dairy buffaloes

The apparent digestibility of animals is closely related to the type of diet. The degradation degree of roughage in livestock rumen is different due to the different quality of roughage in the diet. The apparent digestibility of DM, CP, and OM are affected by the contents of CP and CF in the roughage itself. In this experiment, the digestibility of CP is higher in the A diet with high CP content, while the low CP content of R and S diets results in low CP digestibility due to the lack of digestible carbohydrates and limited microbial protein synthesis. Studies have shown that the crude protein digestibility of the diets was significantly higher in the high CP group than in the low CP group [22], and the results of the present study are consistent with this.

The apparent digestibility of NDF and ADF reflects the dietary fiber utilization capacity of ruminants. In this experiment, the NDF, ADF, and ADL in the K diet and R diet were higher, and the apparent digestibility of NDF, ADF, and ADL in the A diet was lower. NDF digestibility decreased with the increase in dietary protein level. Crop by-products such as wheat straw, straw, and corn cob are rich in fiber and are important sources of roughage for ruminants [23]. The lack of available carbohydrates and proteins in such roughage, coupled with the low digestibility of some tissues of straw forage, restricts rumen fermentation, leading to a decrease in feed intake and feed digestibility of ruminants. The apparent total tract digestibility of nutrients in this study is in agreement with those reported findings for similar forage types [24]. Based on the analysis of the above results, this study found high digestibility of various roughage nutrients in dairy buffaloes. This demonstrates the roughage tolerance characteristics of buffaloes.

### Effects of different roughage on rumen fermentation parameter of dairy buffaloes

Rumen fluid pH reflects rumen fermentation and the internal environment [25]. Rumen pH should be maintained at 6.60–6.80 to maintain a suitable fiber digestion environment [26]. In this study, rumen pH ranged at 6.26 and 6.76, which is within the normal range. O diet had a higher pH compared to the W diet, likely due to increased lignification and physically effective NDF content, increased rumination, chewing times, and salivation, resulting in higher rumen pH. W diet has low pH due to the presence of lactic acids. Rumen fluid pH decreases with higher dietary NFC levels [27]. Diets with high NFC contain fermentable carbohydrates, rapidly fermented by rumen microorganisms and produce a large amount of VFA and organic acids, thus reducing rumen pH. In this experiment, the W diet had the highest NFC content, had the lowest pH. Increasing rapidly fermented starch in the diet increases rumen VFA production, exceeding absorption and cushioning capacity, leading to decrease in rumen pH [28]. In this experiment, the W diet had lower pH compared with other experimental groups, supporting this view.

NH_3_-N concentration in rumen fluid is the end product of rumen fermentation of nitrogen-containing compounds such as feed proteins, which serve as raw material for synthesizing microbial body proteins by rumen microorganisms. It is also an important indicator of the rumen environment [29]. the rumen ammonia nitrogen concentration of cows varied significantly with different roughage types [30]. In this experiment, NH_3_-N concentration in rumen fluid increased with the dietary CP content. The lowest concentration was only 8.57 mg/dL in the R diet, which may be influenced by changes in dietary CP content, nitrogen utilization rate, and roughage degradation by microorganisms [31, 32]. Rumen NH_3_-N gradually decreased up to 12 h after feeding [33]. After 2 h of feeding, the NH_3_-N content of the ruminants gradually decreased with time. In this experiment, rumen fluid collection occurred 2 h after ingestion, and the NH_3_-N concentration in each group was relatively high.

The concentration and proportion of rumen VFA were mainly affected by dietary composition. Generally speaking, roughage has higher content of cellulose, hemicellulose, and lignin, resulting in a higher proportion of acetic acid produced by rumen fermentation [34]. As the quality of roughage improves, non-fiber carbohydrate content increases, the concentration of acetic acid decreases and the concentration of propionic acid increases, leading to a decreasing trend in the acetic acid/propionic acid ratio. In this experiment, the acetic acid/propionic acid value ranged from 3.05 to 4.47, which may be uniformly high due to the low propionic acid content caused by the low proportion of concentrate in the diet [35]. The TVFA content in the W diet and A diet was the highest, with values of 74.48mmol/L and 73.85mmol/L, respectively. These values were 19.32mmol/L and 18.69mmol/L higher than that in the straw hay diet group (55.16mmol/L). The results showed that feeding whole corn silage and alfalfa hay can provide more energy for ruminants, especially by increasing the propionic acid content, which benefits fat accumulation in ruminants. Furthermore, the study showed that dietary NFC content has a significant effect on rumen TVFA production. The TVFA concentration of the A diet and W diet was higher in this study, which may be related to the non-structural carbohydrates contained in alfalfa hay and whole corn silage.

### Effects of different roughage on rumen bacteria of dairy buffaloes

A large number of rumen microorganisms inhabit the rumen of ruminants. Numerous studies have established a connection between the rumen microbiome and influential factors such as feed utilization rate [36], diet [37, 38], animal type [39, 40], animal age [41], and geographical location of animals [42]. Among these factors, diet is considered the most critical. Microorganisms impact rumen fermentation and animal performance through synergistic interaction. The core microbiota of cattle rumen is commonly believed to comprise Firmicutes (particularly Ruminococcus and Butyrivibrio) and Bacteroidetes (especially Prevotella), along with some less abundant groups [43]. The relative abundance of bacteroidetes varied with fat and protein intake. Firmicutes and bacteroidetes were the representative flora in the gastrointestinal tract, and bacteroidetes dominated the rumen with a relative abundance of 59% [44]. *Prevotella* in bacteroidetes is an important starch-degrading bacterium in the rumen. The results showed that the relative abundance of *prevotella* 1 ranged from 30.17 to 45.75%, which was the largest relative abundance among bacteroides.

Most firmicutes are gram-positive bacteria, and many members are beneficial bacteria, with a relative abundance of about 30% in rumen fluid [45]. In this experiment, the relative abundance of firmicutes was maintained between 14.29% and 21.86%, which is consistent with the abundance range indicated by previous researchers [46]. In this experiment, the lowest relative abundance of firmicutes appeared in the S diet, and the highest abundance appeared in the O diet.

Candidate phyla radiation (CPR), also known as patescibacteria is a unique type of bacteria. Brown found in the study of groundwater microbial communities [47]. CPR bacterial cells have a special form and are the smallest known bacterial family on Earth. This study found the bacteria in rumen fluid, possibly, because the buffaloes were drinking water from deep wells, allowing CPR bacteria from the groundwater to enter the rumen during consumption. Spirochaeta bacteria are abundant in the rumen of ruminants, and they can account for 1-6% of the total number of living organisms in the rumen. In this experiment, the relative abundance of spirochaetes ranged from 0.44 to 1.43%. The relative abundance of spirochaetes is also sensitive to roughage.

The Proteobacteria is the main bacterial phylum in the rumen. A large number of studies have indicated that proteobacteria is the third most abundant bacterial phylum in the rumen [48]. The researchers examined rumen microorganisms through 16 S rRNA and Metagenomic detection and found that the relative abundance of proteobacteria was low in the 16 S rRNA detection, which aligned with the findings of this experiment and those of Metzler et al. and Pitta et al. [48, 49].

## Conclusions

DM, OM digestibility was highest in the W diet and lowest in the sugarcane shoot silage (S diet). CP digestibility was highest in the alfalfa hay (A diet) and king grass (K diet) and lowest in the sugarcane shoot silage (S diet) and rice straw hay (R diet).

The Rumen pH of the oat hay (O diet) was significantly higher than that of the whole corn silage (W diet). Rumen fluid NH_3_-N concentration increased with the increase of CP. The concentration of total volatile fatty acids in the rumen decreased with the increase of NDF but increased with the increase of NFC.

The relative abundances of Bacteroidetes, Firmicutes, and Spirochaetes were 57.03-74.84%, 14.29-21.86%, and 0.44-1.43% in roughage groups with different quality. Bacteroidetes were mainly *Prevotellaceae1* and *Rikenellaceae* RC_gut_group with relative abundances of 30.17-45.75% and 3.23-7.82% respectively. The relative abundance of Patescibacteria and Spirochaetes decreased with improved roughage quality.

## Data Availability

The data presented in the study are deposited in the NCBI repository, accession number PRJNA1001128.
